# Dentigerous Cysts in Children: Clinical, Radiological, and Healing Aspects

**DOI:** 10.3390/medicina60071133

**Published:** 2024-07-14

**Authors:** Maria Cristina Langă, Diana Florina Nica, Virgil-Florin Duma, Rodica Elena Heredea, Cosmin Sinescu

**Affiliations:** 1 Faculty of Dental Medicine, “Victor Babes” University of Medicine and Pharmacy of Timisoara, 2 Eftimie Murgu Square, 300041 Timisoara, Romania; langa.cristina@yahoo.com (M.C.L.); minosinescu@yahoo.com (C.S.); 2 3OM Optomechatronics Group, Faculty of Engineering, “Aurel Vlaicu” University of Arad, 2 Elena Dragoi Str., 310130 Arad, Romania; 3 Department of Measurements and Optical Electronics, Faculty of Electronics, Telecommunications, and Information Technology, Polytechnic University of Timisoara, 2 Vasile Parvan Ave., 300223 Timisoara, Romania; 4 Center of Research and Development for Mechatronics, National University of Science and Technology POLITEHNICA Bucharest, 060042 Bucharest, Romania; 5 Department of Pathology, “Louis Turcanu” Children’s Clinical Emergency Hospital, 300041 Timisoara, Romania; heredea.rodica@yahoo.com

**Keywords:** dentigerous cysts, cystic lesions, odontogenic cysts, pediatric patients, surgery, radiography, therapeutic algorithms, bony healing, cone beam-computed tomography

## Abstract

*Background and Objectives*: Dentigerous cysts are one of the most frequent pathologies associated with unerupted or impacted teeth. Such cysts show a male predilection and a preference for the mandibular region. Also, they commonly occur in the second and third decades of life, with only 9% occurring in the first decade. The aim of this work is to apply and study the therapeutic algorithms developed for dentigerous cysts and their outcomes, from the early diagnostic stage to the complete healing phase of pediatric patients diagnosed with this medical condition. *Materials and Methods*: The study included 19 pediatric patients diagnosed with dentigerous cysts who underwent the enucleation and extraction or conservative attitude of the associated tooth. The bony healing was also followed-up 9 months after the surgery. *Results*: A higher incidence in the posterior area of the mandible and maxilla was observed, as well as a higher incidence in boys. The 9 months postoperative radiographic assessment showed that the bony defects were completely healed. *Conclusions*: A thorough understanding of the nature of the lesion backed by a good clinical history and by state-of-the-art radiographic and radiologic examinations can go a long way in helping the surgeon to choose the correct therapeutic approach and to ameliorate the medical condition in the best long-term interest of the young patient. The considered dentigerous cyst cases demonstrated that an early diagnosis and treatment of this pathology is followed by a responsive treatment.

## 1. Introduction

Dentigerous cysts are one of the most frequent pathologies associated with unerupted or impacted teeth. This medical condition shows a male predilection and a preference for the mandibular region [1]. Also, the third molar of the mandible is the most commonly affected tooth [2], followed by the maxillary canine, the maxillary third molar, and the mandibular second premolar [3]. Approximately 95% of all dentigerous cysts involve the permanent dentition, while the other 5% are associated with supernumerary teeth [4]. Dentigerous cysts most commonly occur in the second and third decades of life, and only 9% occur in the first decade [3,5].

These cysts often show no symptoms, and they are generally detected by a radiographic examination [6]. Pain, swelling, tooth mobility, and tooth displacement may occur when the cyst reaches a considerable size [7]. The presence of dentigerous cysts can cause severe consequences, such as pathological bone fractures and facial asymmetry [2].

From a radiographic point of view, dentigerous cystic lesions often present a unilocular radiolucent area around the crown of the impacted tooth, surrounded by a well-defined corticated border [6,8]. They may appear radiographically similar to an odontogenic keratocyst or to unicystic ameloblastoma [2]. Large dentigerous cysts may appear multilocular, resulting in a radiographic appearance that is comparable to other pathologies [9].

From a histopathological point of view, dentigerous cysts usually present a nonkeratinized stratified squamous epithelium with occasional elongated interconnecting rete ridges. A variable number of chronic inflammatory cells may be observed in the underlying connective tissue [4].

Dentigerous cysts can be treated by enucleation or marsupialization. The treatment decision considers different criteria, including size, location, removal of unerupted tooth, and follow-up possibilities [10]. The wisdom teeth are usually removed together with the cyst. However, other teeth, such as canines in the maxilla and premolars in the mandible, should be maintained to secure the continuity of the dental arches [3]. Therefore, the treatment regarding these cysts should compare the risks of removal and the benefits of tooth preservation. Surgical marsupialization or decompression are the two most conservative treatment options described in the literature for the management of dentigerous cysts. However, the removal of the cystic lesion and the extraction of the unerupted tooth are the main treatments for preventing the recurrence of the cystic lesion [2,6].

The aim of the present work is to present therapeutic algorithms developed for dentigerous cysts, from the early diagnosis stage to the complete healing phase of pediatric patients diagnosed with this medical condition. Such an algorithm of treating dentigerous cysts during childhood consists of fine needle aspiration cytology (FNAC) and enucleation. Applying FNAC as the first step allows for getting closer to the histopathological diagnosis prior to surgery. Thus, from the beginning, one could apply the most conservative surgical approach, as it is the scope of the present study. The null hypothesis (H0) of this work posits no importance of the diagnosis and treatment approach of the considered medical condition.

## 2. Materials and Methods

In this study, we examined the characteristics of 19 dentigerous cysts developed in pediatric patients, performed the treatment, and followed-up the bony healing at nine months after surgery. The size, shape, and position of the cysts, as well as the relationship with the causal tooth were initially analyzed using panoramic radiography and cone beam computed tomography (CBCT). A CRANEX 3DX X-ray radiography system (Soredex, Tuusula, Finland) was utilized for all imaging investigations. For each case, the best treatment option was discussed. The defect ossification and the eruption process of the causal teeth was evaluated after the performed treatment using CBCT.

### 2.1. Clinical Subjects

A prospective monocentric observational study was carried out between March 2021 and February 2024 at the Department of Oral and Maxillofacial Surgery of the “Victor Babes” University of Medicine and Farmacy of Timisoara. This study was carried out according to the Declaration of Helsinki and was approved by the Ethics Commission for Scientific Research, following the Ethical protocol of the University, with the CECS Approval no. 09/02 March 2018 and no. 83/19 December 2022 for its revision. Informed consent was obtained from the parent or legal guardian of each of the enrolled patients.

The 19 pediatric patients were included in the study because of their diagnoses with dentigerous cysts, as presented in Table 1. They underwent the enucleation and extraction of the associated tooth, or a conservative approach. In order to have a presumptive diagnosis, prior surgery, an FNAC, was performed for all the considered cases by using a 21–25 G needle and a 20 mL syringe. The needle was inserted into the lesion through the resorbed vestibular plate and the aspirate was smeared onto glass slide, fixed in 95% ethyl alcohol for hematoxylin and eosin staining, and air dried for Romanowsky staining.

### 2.2. Clinical Cases

#### 2.2.1. Case 1

**Figure 1:**
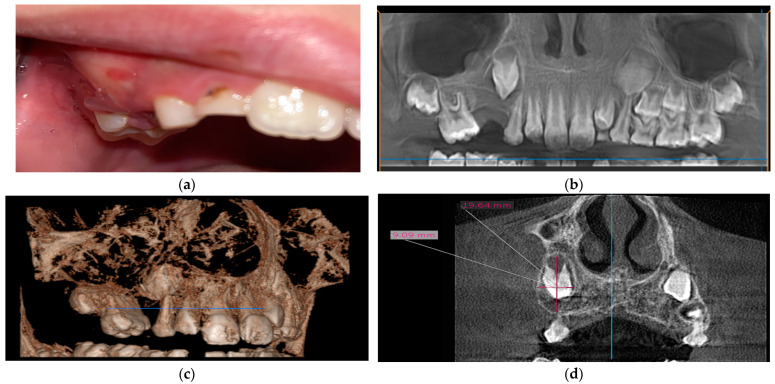
Case 1’s initial preoperative images: (**a**) intraoral view; (**b**) CBCT-panoramic view; (**c**) CBCT reconstruction; (**d**) cross-sectional view with measurements.

A nine-year-old boy, without any relevant medical history, was referred to our department because of a persistent painless maxillary swelling in the right cheek region. His deciduous right upper molars had been extracted at the age of 7 due to severe decay complications. The first premolar, which erupted earlier, was also extracted at the age of 8, and the second premolar was congenitally absent. An intraoral examination revealed a volume augmentation under normal alveolar mucosa in the region of the right upper canine (Figure 1a). The palpation of the deformed region showed a painless ‘eggshell cracking’ feeling, along with a partially elastic consistency. This case is relevant due to the child’s young age and the conservative approach that allowed for maintaining the causal tooth.

The CBCT examination showed an ovate radiolucency surrounding the upper canine, adjacent to the apex of the deciduous canine and to the middle wall of the right maxillary sinus (Figure 1b). The buccal cortical plate appeared thinned and expanded in the cranial cystic area, as well as resorbed in the caudal direction (Figure 1c). The length of the vertical major axis of the radiolucent area was 19.4 mm (Figure 1d). Based on the clinical and radiological findings, the initial diagnosis was dentigerous cyst.

The surgical intervention was made under general anesthesia. Additionally, local anesthesia (using 4% articaine and 1:100,000 epinephrine) was utilized with a direct troncular technique to anesthetize the infraorbital nerve. Also, an anesthetic infiltration on the palatal side for the naso-incisive and greater palatal nerve was carried out. A trapezoidal mucoperiosteal flap was elevated, showing the apertured buccal plate. Ostectomy was performed with a round tungsten carbide bur under saline irrigation, preserving the bone as much as possible. The complete enucleation of the lesion was performed with care around the entire length of the canine in order to perform an accurate surgical cleaning. The wound was sutured, and the healing was further monitored for 21 days. The lesion was immersed in a 10% formaldehyde solution and sent for histopathological examination.

**Figure 2:**
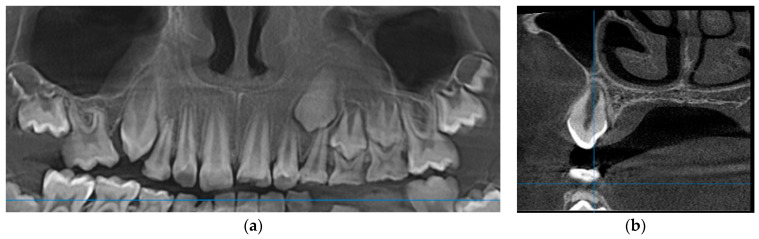
Case 1’s CBCT performed 9 months after the surgery: (**a**) panoramic view; (**b**) cross-sectional view.

Clinical and radiological re-evaluation were performed 9 months after the surgery. The complete healing of the surgery site and the eruption of the canine were assessed. Panoramic and cross-sectional view on a re-evaluation CBCT showed the complete repair of the hard tissue defect (Figure 2).

#### 2.2.2. Case 2

A 9-year-old male patient was referred to our department for evaluation of the persisting swelling in the right mandible vestibule. This case is relevant due to the difficulties in the diagnosis process, as the lesion could only be seen through 3D radiography.

**Figure 3:**
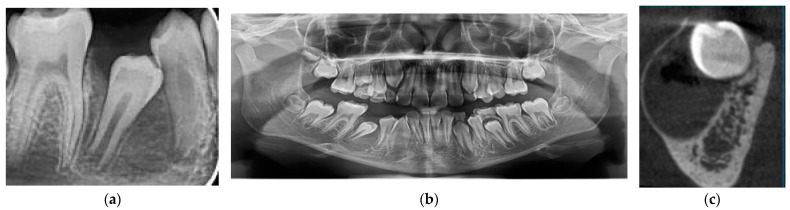
Case 2’s initial preoperative images: (**a**) retro alveolar radiography; (**b**) panoramic view showing the discrete encircling radiolucency; (**c**) cortical expansion.

The clinical examination revealed a hard submucosal deformation of the vestibular cortical plate, covered by an unmodified mucosa in the region of the second premolar. The retro alveolar radiography of the affected region showed an impacted second premolar partially surrounded by a fine radiolucency (Figure 3a). Further panoramic exposure allowed for a better evaluation of the impacted tooth encircled by a round and discrete radiolucency (Figure 3b). An accurate CBCT in the cross-sectional view showed a perfectly round, 2.5 cm diameter radiolucency containing the tilted second premolar crown within the mandible, with an expanded external cortical plate (Figure 3c).

**Figure 4:**
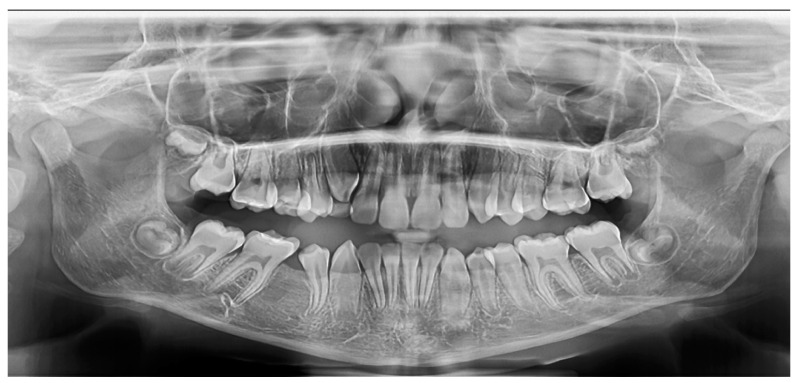
Case 2: complete bone healing after 9 months.

Based on the clinical and radiographic evaluation, a preliminary diagnosis of dentigerous cyst was made. Due to the tilted position of the included second premolar, the decision of extraction was made, together with a complete removal of the cyst. The histopathological diagnosis was also dentigerous cyst. A radiological check was performed nine months after the surgery, showing a complete healing with no resorption at the level of the neighboring teeth (Figure 4).

#### 2.2.3. Case 3

**Figure 5:**
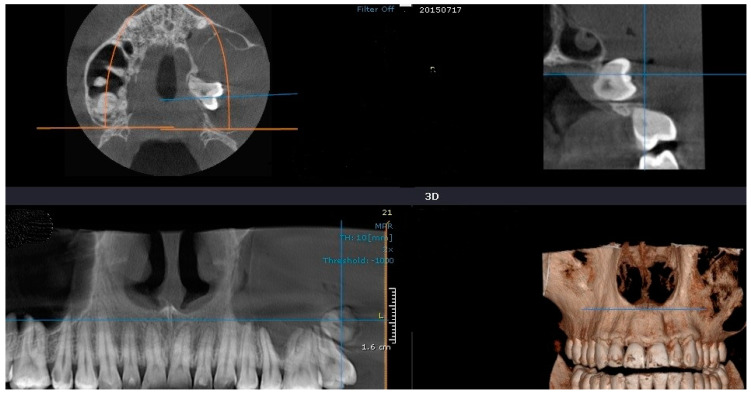
Case 3: A 6.7 cm diameter cyst occupying the left maxillary sinus was diagnosed.

**Figure 6:**
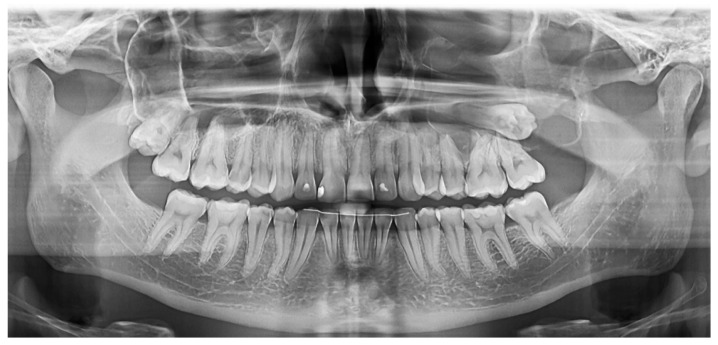
Case 3: The panoramic view of the initial status.

A large cystic lesion was diagnosed in an 18-year-old patient within the left maxillary sinus, with an expansion and a partial resorption of the vestibular plate, caused by an impacted upper third molar (Figure 5 and Figure 6). This case is relevant because it was the largest treated lesion interfering with the maxillary sinus.

**Figure 7:**
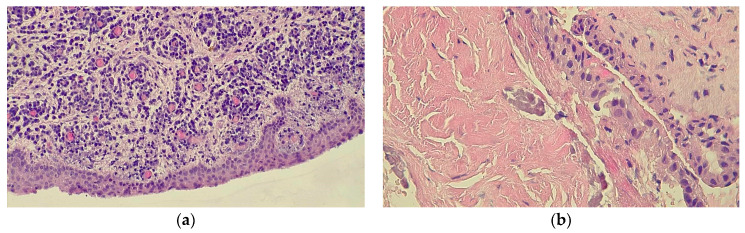
Case 3: The histopathological aspect in the initial (diagnosis) stage: (**a**) nonkeratinized stratified squamous epithelium; (**b**) cholesterol clefts.

In the histopathologic investigations, the hematoxylin and eosin-stained sections showed a nonkeratinized stratified squamous epithelium, with rete ridges proliferating into the connective tissue stroma and cholesterol clefts (Figure 7). Therefore, the histopathological diagnosis was also dentigerous cyst.

The surgery was performed with a conservative approach for the sinus mucosa, performing enucleation and odontectomy on 2.8 cm of the cyst’s membrane. The buccal cortical plate corresponding to the first and second left upper molars was almost completely resorbed at the time of patient presentation, and the surgical approach also required enucleation of the cyst that was in contact with the periapical space.

**Figure 8:**
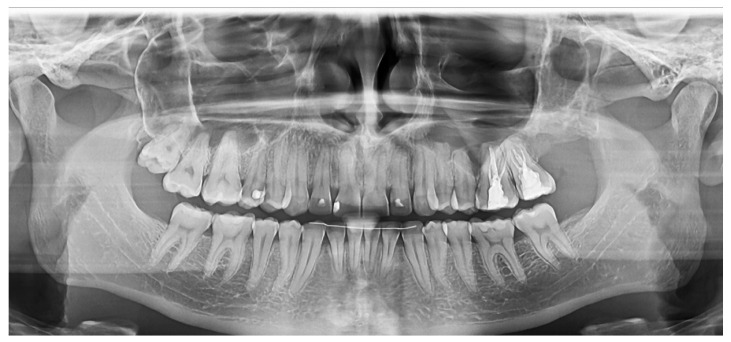
Case 3: panoramic view obtained after the 9 months follow-up after surgery, showing a complete regeneration of the posterior maxilla.

A panoramic view was taken nine months after the surgery, showing a complete regeneration of the posterior maxilla wall, with no bone loss around the first and the second left upper molars partially involved with the cyst (Figure 8).

Such outcomes, obtained for all the 19 considered patients in this work necessitates the rejection of the null hypothesis (H0), which posited no importance of the diagnosis and treatment approach of this medical condition. Instead, the alternative hypothesis (H1) is accepted, suggesting that this pathology (which, even if it is rare in the first decade of life, represents a challenging medical problem), when diagnosed early and accurately, is followed by a responsive treatment.

## 3. Results

In total, 19 children with dentigerous cysts in the maxilla and in the mandibular region who fulfilled all the inclusion criteria were selected for the present study. The patients’ ages ranged from 8 to 18 years. Primary wound closure was achieved at the moment of surgery. During the healing time, one patient developed an oroantral communication that was closed in a second surgery without associated sinusitis. The 9-months-post-operative radiographic assessment showed that the bony defects were completely healed. All the osseous defects that resulted after cystectomies healed completely, as demonstrated on a CBCT performed 9 months after the surgery.

From a total of 19 patients presenting the pathology of interest (with ages between 8 and 18 y/o), there were 12 boys and 7 girls; therefore, 63% were boys, and only 37% were girls, suggesting a higher incidence of this medical condition in boys. From the total number of cases, 11 were cystic lesions of the mandible, for 8 boys and only 3 girls, meaning 73% boys and 27% girls were affected by mandibular cystic lesions. Eight cases were reported at the maxilla, with an equal number of four per each sex, 50% percentage for each sex.

**Figure 9:**
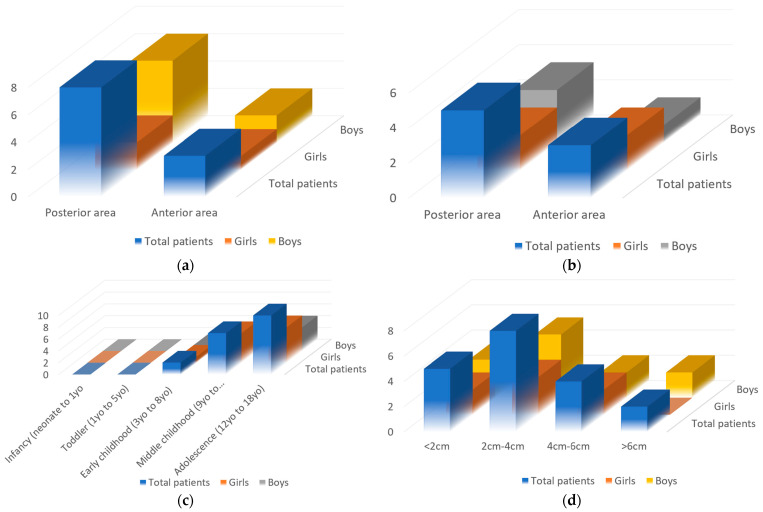
Statistic of the patients included in the study, with regard to their sex, for (**a**) mandible and (**b**) maxillary cystic lesions, also referring to (**c**) the age of the patients and (**d**) the lesion size.

Regarding the location in the bone, for the maxilla, in five cases, the cystic lesion was located in the posterior area (for 3 boys and only 2 girls), while three cases were located in the frontal region (for 1 boy and 2 girls). At the mandible, from a total of 11 cysts, 8 were related to molars or premolars, 6 boys and 2 girls, and only 3 to canines, 2 boys and 1 girl. Therefore, a higher incidence in the posterior area and also a higher overall incidence is observed in boys (Figure 9).

From the reported cases, the patients’ ages are between 8 and 18, with an age average of 12.57. Therefore, no cases were reported in the infancy stage (neonate to 1 y/o) or toddler stage (1 y/o to y/o). In the childhood stage, 9 cases were reported, 2 in the early childhood stage (3 y/o to 8 y/o), for 1 boy and 1 girl, and 7 in the middle childhood stage (9 y/o to 11 y/o), 5 boys and 2 girls. In the adolescence or teenage stage, from a total of 10 patients presenting cystic lesions, 6 were boys and 4 were girls (Figure 9c).

Regarding the size of the cystic lesions, there were 5 patients with lesions under 2 cm in diameter (3 boys and 2 girls), 8 patients with lesions between 2 cm and 4 cm in diameter (5 boys and 3 girls), 4 patients with cysts between 4 cm and 6 cm (2 boys and 2 girls), while 2 patients had lesions bigger than 6 cm in diameter (both of them boys) Figure 9d.

Regarding the management of the impacted tooth, we obtained the preservation, a complete eruption, and a proper position of the arch in 3 cases involving a canine. In all the other cases, because of the deep position and misalignment, the causal tooth was extracted during the surgery.

## 4. Discussion

The aim of this paper is to present the therapeutic algorithms developed for pediatric patients diagnosed with dentigerous cyst, considered from the early diagnostic stage to the complete healing phase, the latter monitored 9 months after surgery.

The dentigerous cyst belongs to the developmental category and is usually related to an impacted tooth [11]. It is the second most common odontogenic cyst after the radicular one. A cystic lesion diagnosed in children or adolescents is developed around the crown of an unerupted tooth and most likely represents a dentigerous, also called follicular, cyst. Its origin is from the dental sac or dental follicle of the causal tooth with fluid accumulation [8].

The highest prevalence of a dentigerous cyst is in the first and third decade of life [12]. The prevalence in the first decade of life has been reported only in a few studies [13,14]. Isolated cases of the association of dentigerous cysts with primary teeth have been reported [15]. Our work has highlighted the importance of the dentigerous cystic pathology in children, presenting the algorithm of diagnosis and rational treatment.

In the present study, we considered 19 patients diagnosed with dentigerous cysts. In almost half of these pediatric patients, this specific pathology was found in the adolescence age. We found a male predilection, while the most prevalent anatomical location was in the posterior mandible, followed by posterior maxilla. This was consistent with the data in a review of the literature [16].

The ortopanoramic aspect of a dentigerous cyst is often a unilocular radiolucent area surrounding the crown of the impacted tooth with fine corticated borders [8,16]. Several other entities that have a similar radiological aspect must be considered as a differential diagnosis, including radicular cyst, residual cyst, idiopathic bone cavity, Pindborg tumor, ameloblastic fibroma, unicystic ameloblastoma, odontogenic keratocyst, and adenomatoid odontgenic tumor [17]. Large cysts may have a multilocular feature, suggesting an ameloblastoma, a odontogenic keratocyst, or an odontogenic myxoma [8,18]. All the mandibular cases in our study appeared as unilocular radiolucency surrounding the neck of the crown of an unerupted tooth, giving the first choice of diagnosis as a dentigerous cyst.

In our *5 posterior maxillary cases*, because of the large cystic cavity and the overlap of the maxillary sinus, which provided a multilocular radiological aspect, an aggressive tumor had to be considered. In these cases, the differential diagnosis included ameloblastic fibroma and keratocystic lesions.

**Figure 10:**
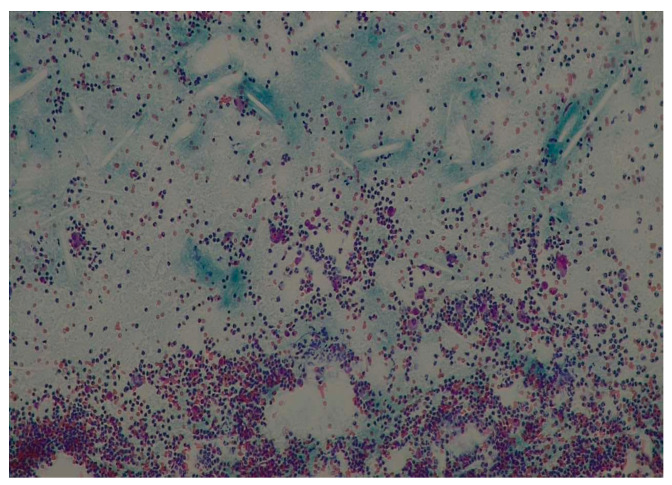
A representative example of the cytology of a dentigerous cyst.

The histopathological diagnosis is crucial in establishing a correct surgical attitude, especially in our considered pediatric cases (as presented as an example in Figure 10), in order to exclude the malignant pathologies with similar initial radiological appearances, such as squamous cell carcinoma, verucous carcinoma, or mucoepidermoid carcinoma [19]. In order to have a presumptive diagnosis, prior to surgery, an FNAC was performed for all cases using a 22 G needle and a 20 mL syringe. The needle was inserted into the lesion through the resorbed or thinned vestibular plate in corroboration with radiological findings, and the aspirate was smeared onto a glass slide and fixed in 95% ethyl alcohol for hematoxylin and eosin staining. For the considered patients, the FNAC was performed on a different day, 1 or 2 days before the surgery.

The FNAC proved to be fast, non-invasive, safe, and easy to perform, with a high sensitivity (94.7%) and specificity (100%), as well as with a diagnostic accuracy of 97.3% [20]. Therefore, the FNAC allowed us to rule out entities such as radicular cyst, ameloblastoma, odontogenic myxoma, odontogenic keratocyst, and malignant tumors [21]. The aspirate provided pale yellow fluid containing squamous cells in reduced number and macrophages. This procedure supported us for a rational surgical planning with a minimally invasive procedure and with maximum bone preservation.

The techniques that proved to be effective for the treatment of dentigerous cysts are as follows: decompression, marsupialization, and complete surgical enucleation [22,23]. Decompression and marsupialization are mostly utilized as conservative approaches in cases of a proper pre-operatory diagnosis. They have the advantage of a minimally invasive procedure with the protection of neighboring anatomical structures, together with the preservation of the causal tooth [24]. In our cases, we achieved a *conservative approach to cyst-associated teeth in 3 out of a total of 19 cases*. All the involved teeth were canines, two in the maxillary and one in the mandible. A complete removal of the cystic membrane was performed. This technique was described by Hauer et al. [25] in a study performed on 15 unerupted, developing teeth associated with a dentigerous cyst, and it has the major advantages of a certain diagnosis and the maintenance of the affected tooth on the arch. *For the other cases, we performed the complete removal of the cystic membrane and extraction of the impacted tooth due to insufficient space on the arch or unfavorable axis*.

Regarding the conservative approach, in the management of the related tooth, we obtained the complete root developing together with a functional placement on the arch in 3 out of a total of 19 cases. This was possible because of good bone healing and also because of the stage of dental development. Our results seem to be in good agreement with those reported by Nahajowsky et al. [3], who showed that the impacted teeth in cases of dentigerous cysts can reach their functionality if their root development is less than or equal to half of a complete formation [2,26]. In our cases, the bone regeneration was monitored during the healing time with a 3D imaging capture 9 months after the surgery. In all cases, a complete bone filling of the cyst defect was found. One must highlight that the healing follow-up has a crucial importance in dentigerous cystic pathology because of their potential of recurrence with attaining larger size and the potential of neoplastic change [27,28].

**Figure 11:**
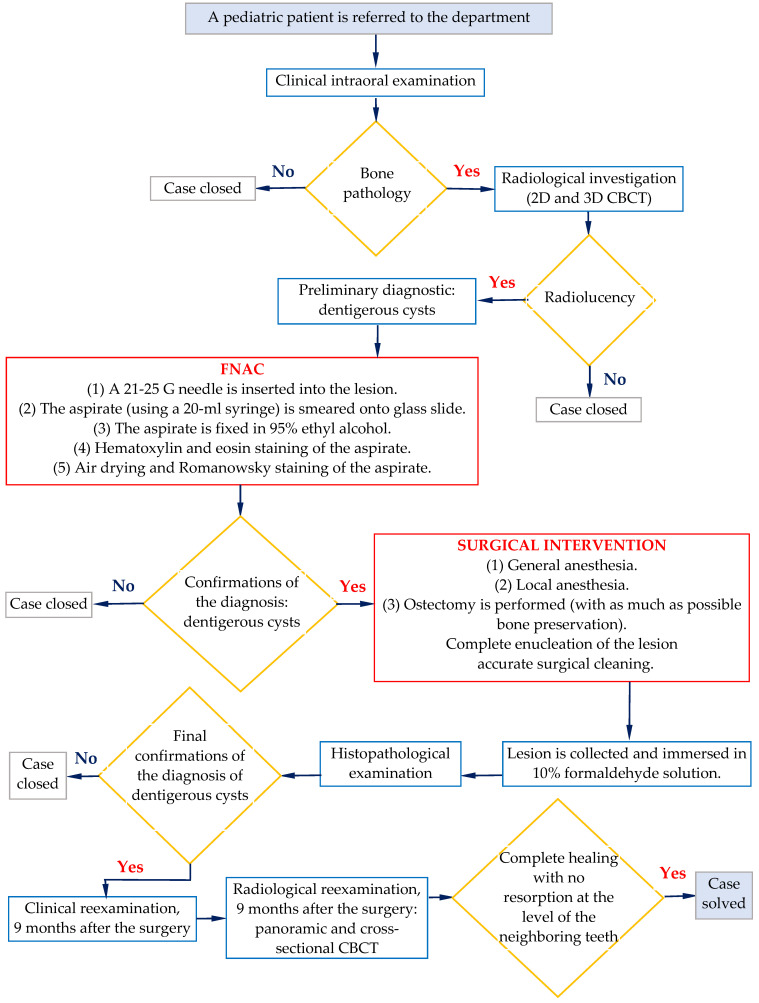
Developed therapeutic algorithm for the treatment of dentigerous cysts in pediatric patients.

Therefore, this study has highlighted the following: (i) a large number of pediatric patients diagnosed with dentigerous cysts, despite the small percentage of patients diagnosed with this pathology in the first decade of life, which is about 9% according to the literature [3,5]; (ii) the efficiency of the work protocol, which included FNAC in all the cases in order to have an accurate diagnosis prior to surgery; (iii) the possibility to manage the situation in order to preserve the causal tooth in 3 cases and to completely remove the cystic membrane to favor the eruption of important teeth such as the canine; (iv) the follow-up 9 months post-surgery, which showed that the bony defects were completely healed and that no signs of recurrence were observed. The developed algorithm that is proposed as a result of this work is presented in Figure 11 to summarize the therapeutic procedure.

The limitations of the present study may include the number of patients diagnosed with the considered medical condition. In this respect, our group’s work is planned to continue on a larger pool of patients in the following years. Regarding the utilized imaging method, while panoramic radiography and CBCT scans are the gold standard for the assessment of dental conditions such as those considered in the study, future work in our group will consider using optical coherence tomography as well, both for correlated investigations [29] and for optimizing the characteristic parameters of dental radiographies [30].

## 5. Conclusions

The management of dentigerous cysts in children represents a challenge for dentists, maxillofacial surgeons, anatomo-pathologists, and orthodontists. The present study is an argument for how a thorough understanding of the nature of the lesion backed by a good clinical history and state-of-the-art radiography can go a long way in helping the surgeon to choose the correct therapeutic approach. Also, the study pointed out how to ameliorate the medical condition in the best long-term interest of a young patient. Our considered dentigerous cyst cases demonstrated that this pathology, even if it is rare in the first decade of life, represents a challenging medical condition. However, an early and accurate diagnosis of these lesions is followed by a responsive treatment.

## Figures and Tables

**Table 1:** Overview of the 19 patients and of the characteristics of their approached medical condition.

Nr.	Age	Sex	Mandible/Maxilla	Causal Tooth (Cyst Localization)	FNAC	Size of the Cystic Lesion (cm)
1	16 y/o	M	Mandible	4.8 (right mandibulary third molar)	yes	1.9
2	11 y/o	M	Maxilla	1.5 (right maxillary second premolar)	yes	4.52
3	13 y/o	F	Maxilla	2.3 (left maxillary canine)	yes	1.73
4	9 y/o	M	Mandible	4.5 (right mandibulary second premolar)	yes	2.5
5	6 y/o	F	Maxilla	1.2 (right maxillary lateral incisor)	yes	1.82
6	14 y/o	M	Mandible	3.5 (left mandibulary second premolar)	yes	2.25
7	10 y/o	M	Mandible	4.2 (right lateral incisor)	yes	2.11
8	13 y/o	F	Maxilla	2.5 (left maxillary second premolar)	yes	4.7
9	14 y/o	F	Mandible	4.5 (right mandibulary second premolar)	yes	2.82
10	9 y/o	M	Maxilla	1.3 (right maxillary canine)	yes	1.94
11	7 y/o	M	Mandible	4.2 (right mandibulary lateral incisor)	yes	1.5
12	16 y/o	M	Mandible	3.8 (left mandibulary third molar)	yes	6.1
13	11 y/o	M	Mandible	4.5 (right mandibulary second premolar)	yes	3.65
14	18 y/o	M	Mandible	3.8 (left mandibulary third molar)	yes	3.3
15	9 y/o	F	Mandible	3.4 (left mandibulary first premolar)	yes	3.1
16	18 y/o	F	Maxilla	1.8 (right maxillary third molar)	yes	4.8
17	18 y/o	M	Maxilla	2.8 (left maxillary third molar)	yes	6.7
18	16 y/o	M	Maxilla	1.8 (right maxillary third molar)	yes	5.1
19	11 y/o	F	Mandible	3.3 (left mandibulary canine)	yes	2.3

## Data Availability

Data supporting the reported results can be obtained from the corresponding author.
